# Evidence-Based Practice in Rehabilitation of Myasthenia Gravis. A Systematic Review of the Literature

**DOI:** 10.3390/jfmk5040071

**Published:** 2020-09-27

**Authors:** Bruno Corrado, Benedetto Giardulli, Massimo Costa

**Affiliations:** 1 Department of Public Health, University Federico II of Naples, Via S. Pansini n.5, 80131 Naples, Italy; benedettogiardulli@gmail.com; 2 Department of Polyspecialistic Medicine, Cardarelli Hospital, Via A. Cardarelli, 80131 Naples, Italy; massimo.costa@aocardarelli.it

**Keywords:** congenital myasthenic syndromes, muscle weakness, myasthenia gravis, neuromuscular junction, physical therapy modalities, rehabilitation

## Abstract

Myasthenia gravis is a rare neuromuscular disorder characterized by muscle weakness and fatigue. This review analyzes the most recent evidence regarding the effectiveness and safety of different rehabilitative approaches to the disease. The review was carried out in accordance with the Preferred Reporting Items for Systematic Reviews and Meta-Analyses (PRISMA) guidelines. A total of 365 articles were found in the main scientific databases. Applying the inclusion/exclusion criteria, 11 studies were admitted to the final phase of the review. Three different rehabilitative approaches were identified: physical training, respiratory training, and balance training. All rehabilitative modalities contributed to enhancing functional outcomes, reducing fatigue, and improving quality of life, but currently none can be recommended over another for the lack of cross-comparative studies. The included studies showed methodological quality from low to fair. Despite the range of rehabilitative interventions available, there is a lack of high-quality evidence. However, this review suggests that a multidisciplinary rehabilitation approach should be recommended to people with myasthenia gravis, and above all, for those with mild to moderate symptomatology.

## 1. Introduction

Myasthenia gravis (MG) is an autoimmune disorder due to a postsynaptic defect of neuromuscular transmission. MG is caused in the majority of patients by autoantibodies directed against the postsynaptic nicotinic acetylcholine receptor (AChR). The annual incidence of MG is 8–10 cases per 1 million persons and its prevalence is 150–250 cases per 1 million persons [1]. MG can affect people of any age, typically starting in women under 40 and men over 60. Muscle specific kinase myasthenia gravis (MuSK-MG) is a rare subgroup of MG affecting mainly women during childbearing years [2].

The clinical manifestation of MG is weakness of skeletal muscles, which increases with fatigue and during the day, often with nearly normal muscle strength in the morning. The muscular weakness can be localized or generalized, and usually is more proximal than distal [3]. Eye and oropharyngeal muscles are often affected, but the distribution of muscle weakness is highly variable [4]. Muscle weakness causes common symptoms of MG that include: fatigue, breathing difficulties, ptosis, diplopia, hypomimia, problems with chewing and swallowing, and dysarthria. Myasthenic crisis is a complication of MG characterized by worsening of muscle weakness, resulting in respiratory failure that requires intubation and mechanical ventilation. MG and thymic neoplasms are frequently associated; one half of cortical thymoma patients develop MG, while 15% of MG patients have thymomas [5,6]. MG has an extensive impact on physical, psychological, and social wellbeing of the patients, causing a reduced health related quality of life (HRQoL): the more severe muscle symptoms and disability, the lower the physical components of the outcome [7,8,9,10].

The diagnostic approach to MG can be difficult and is focused on confirming the clinical diagnosis established by the history and typical examination findings. Point-of-care tests (the ice pack test, alone or combined with sustained upgaze, and the edrophonium test) are sensitive, but they have major limitations due to concerns about excess false-positive results [11]. The most reliable laboratory methods that aid in the confirmation are serologic tests for autoantibodies and electrophysiological studies (repetitive nerve stimulation—RNS, and single-fiber electromyography—SFEMG). MG can be classified in the following main subgroups defined on the basis of clinical, antibody, and thymic features: late onset, early onset, ocular, seronegative, thymoma, lipoprotein receptor–related protein 4 (LRP4), and MuSK [12]. According to the Osserman and Genkins classification, the clinical severity of MG is graded in five stages (I, ocular signs only; IIA, generalized mild muscle weakness; IIB, generalized moderate weakness and/or bulbar dysfunction; III, acute fulminating presentation and/or respiratory dysfunction; IV, late generalized weakness) [13].

Treatment of MG includes acetylcholinesterase inhibitors (e.g., pyridostigmine, edrophonium, and ephedrine), thymectomy, immunosuppressive agents (e.g., prednisone, azathioprine, rituximab, tocilizumab, and mycophenolate mofetil), and short-term immunomodulation with plasma-exchange and intravenous immunoglobulin (IVIG) [14,15,16,17,18].

Rehabilitation is defined as “a set of measures that assist individuals who experience disability to achieve and maintain optimal physical, sensory, intellectual, psychological and social functioning in interaction with their environment” (World Health Organization. World Report on Disability. Geneva, Switzerland: WHO; 2011). It is a complex process of delivering a coordinated interdisciplinary care program, comprising a series of individualized and goal-oriented therapies tailored for specific patient needs. The goal of rehabilitation is to improve functional independence and enhance participation with emphasis on patient education and self-management. In neuromuscular disorders, an effective rehabilitation program can help maximize the patient’s physical and psychosocial functions as well as maintain a patient’s quality of life [19,20,21,22]. Furthermore, an effective rehabilitation program can minimize secondary medical comorbidities, prevent or limit physical deformities, and allow the patient to integrate into society [23,24,25,26].

According to experts’ recommendations, rehabilitation is essential in the management of possible MG complications such as contractures and respiratory failure [27]. However, it is well known that typical MG weakness increases with exercise and repetitive muscle use [28]. It follows that it is not clear whether exercise is beneficial or harmful for patients with MG. Therefore, the role of exercise in the management of these patients remains controversial [29,30]. Because muscle weakness is the main problem, muscular exercise would be valuable if it helped to counteract the loss of muscle tissue and strength.

This systematic review arises from the need to clarify the role of physical therapy in the management of patients with MG, since there is a controversial background. The secondary objective is to identify the most effective rehabilitative approaches to improve functional outcomes and quality of life of MG patients according to scientific literature.

## 2. Materials and Methods

The PICO method [31] was used to formulate the clinical query according to the following parameters:

- Population: MG people
- Intervention: rehabilitation/physical therapy/physiotherapy
- Comparator: inactive MG patients
- Outcome: improvement of functional outcome and quality of life

This systematic review was performed in accordance with the PRISMA reporting guidelines for conducting a systematic review or a meta-analysis of intervention trials [32].

### 2.1. Study Eligibility Criteria and Report Eligibility Criteria

The following study eligibility criteria were applied for this systematic review: (a) patients of any age affected by MG; (b) rehabilitation applied to at least a proportion of the patients; (c) enough data provided for the purpose of the review; (d) no limits applied as to the minimum length of the follow-up. The criteria for the report were as follows: (a) written in the English language; (b) including already published data; and (c) including studies published up until December 2019. Letters, comments, editorials, and practice guidelines were excluded. 

### 2.2. Information Sources

For the purpose of identifying relevant studies, a systematic review of the literature was performed using the following databases: PubMed, PEDro, CINHAL, Cochrane Library, EDS Base Index, and TRIP. Articles were limited to the following study designs: randomized controlled trial (RCT), case-control study, and cohort study. The literature search was conducted by two investigators independently.

### 2.3. Search Strategy

The following search terms were used: ‘myasthenia gravis,’ ‘rehabilitation,’ ‘physical therapy,’ and ‘physiotherapy.’ Table 1 summarizes the full electronic search strategy for the PubMed database.

### 2.4. Study Selection

Eligibility assessment of the selected studies was performed independently by two reviewers in an unblinded and standardized manner. All titles and abstracts were screened and ineligible articles were excluded. Then, the full text of the studies meeting the inclusion criteria were reviewed in detail by the investigators. Disagreements between reviewers were resolved by discussion.

### 2.5. Data Collection Process

Data from original articles were recorded on a data extraction form. The following data were extracted by one investigator and then crosschecked by the other: general information concerning the study (lead author and year of publication), study design, rehabilitation approaches in the experimental group, number of participants, and main results/findings. Disagreements between the two reviewers were resolved by discussion; if no agreement could be reached, a third reviewer would be invited to make a decision.

### 2.6. Methodological Quality and Level of Evidence Assessment Process

Methodological quality and level of evidence were assessed independently by two of the investigators. Methodological quality was assessed using the revised Cochrane Risk of Bias Assessment (RoB 2) tool for RCTs, and the modified Newcastle–Ottawa Scale (NOS) for case-control and cohort studies [33,34]. The level of evidence was assessed using the Oxford Centre for Evidence-Based Medicine (OCEBM)—Levels of Evidence guide [35]. Publication bias was not assessed because of the very small number of selected studies.

## 3. Results

### 3.1. Results of the Search

**Figure 1:**
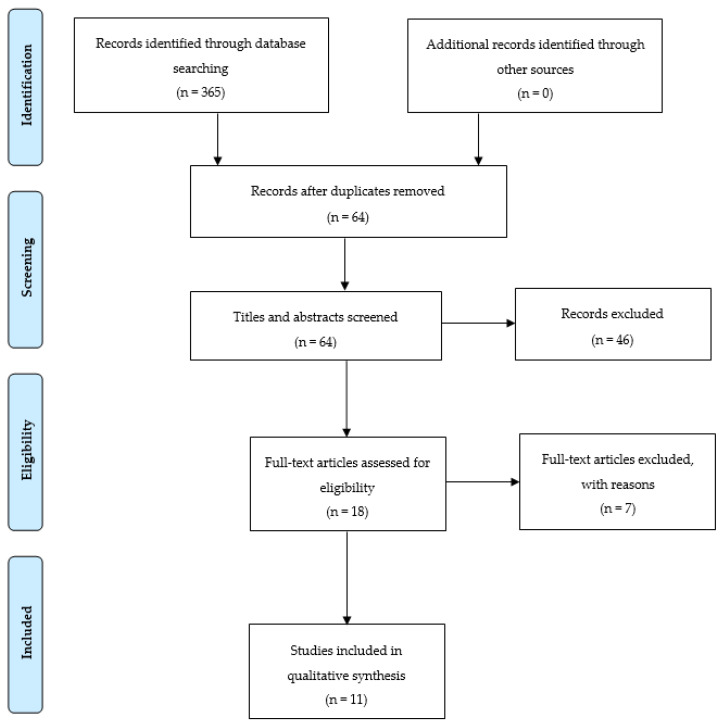
PRISMA flow diagram concerning selection of articles for the review.

A total of 365 potentially relevant studies emerged from the first search in PubMed, PEDro, CINHAL, Cochrane Library, EDS Base Index, and TRIP databases. After duplicates removed (*n* = 301), 64 articles were identified. Therefore, the screening of the titles and abstracts allowed an additional 46 records to be eliminated. The full-text version of a total of 18 records was assessed; seven articles did not meet the inclusion criteria and were excluded with reasons. Eventually, a total of 11 studies were considered eligible. The selected results were classified as follows: two RCTs, one prospective case-control study, and eight cohort studies. From the detailed analysis of these articles, three approaches to rehabilitation in patients with MG were identified: physical training, respiratory training, and balance training. The PRISMA flow diagram used for study selection process is summarized in Figure 1. The details of studies selected for the systematic review are listed in Table 2.

### 3.2. Description of Single Studies

#### 3.2.1. Physical Training

Five of the selected papers belong to this group. The 1993 study by Lohi et al. [36] included 11 patients with mild to moderate MG who followed a strength training program of 30 sessions over 10 weeks. The only positive result was a 23% increase in maximum voluntary muscle force in knee extension on the trained side compared with the untrained one. No adverse effects were registered. 

The aim of the prospective study by Rahbeck et al. (2017) [37] was to establish whether progressive resistance training and aerobic training are feasible and efficient in MG. Fifteen subjects with generalized MG were randomly assigned to 20 training sessions during 8 weeks. No change in Quantitative Myasthenia Gravis (QMG) score was observed in either group. Maximal strength and functional capacity increased in the progressive resistance training group only.

In the prospective pilot study by Westerberg et al. (2017) [38], 10 MG patients with a mild form of the disease performed supervised aerobic and resistance training twice weekly for 12 weeks. Physical exercise was well tolerated, and the Myasthenia Gravis Composite (MGC) score was unchanged. Physical performance-based measures improved while muscle enzymes remained normal.

In their 2018 study, Westerberg et al. [39] evaluated functional skeletal muscle parameters in 11 MG patients, before and after conducting a 12-week supervised physical therapy regimen of aerobic and resistance strength training. After the training program, physical performance-based measures improved as well as the clinical MG composite score. 

The aim of the study by Farrugia et al. (2018) [40] was to investigate whether a combination of physical and psychological therapy would help address symptoms of fatigue in ten MG patients, who have stable disease but residual problematic fatigue. There was a significant improvement in the visual analogue fatigue scale (VAFS) at the end of the program. No clear improvement was noted in the other scales. Three months later, all fatigue scores declined to baseline. 

#### 3.2.2. Respiratory Training

Respiratory training was analyzed in five of the selected studies. Weiner et al. (1998) [41] determined the effects of respiratory muscle training on respiratory muscle strength and endurance in 18 patients with moderate (group A) to severe (group B) generalized MG. The program consisted of both inspiratory and expiratory (group A) or inspiratory alone (group B) muscle training for 30′/day, 6 times a week, for 3 months. The maximal inspiratory muscle pressure, the forced vital capacity, the forced expiratory volume, and the dyspnea index score increased significantly in both groups. 

In the 2005 RCT by Fregonezi et al. [42], 27 patients with stable MG were randomized into training and control groups. The training group underwent a partial-home program consisting of interval-based inspiratory muscle training combined with breathing retraining, three times a week for 8 weeks. The training group improved in maximum expiratory and inspiratory pressures, respiratory muscle endurance, and thoracic mobility compared to control group. 

In their 2007 study, Rassler et al. [43] tested the effect of home-based respiratory muscle endurance training in 10 patients with mild to moderate generalized MG. The training program consisted of normocapnic hyperpnea training at 50–60% of the maximal voluntary ventilation over 4–6 weeks. The training significantly increased respiratory endurance and total ventilatory volume. About 25% of this gain was lost after 3–5 months of detraining.

Aslan et al. (2014) [44] carried out an RCT investigating the effects of respiratory muscle training performed by inspiratory and expiratory threshold loading on pulmonary functions in 26 patients with slowly progressive neuromuscular disease, including MG. Maximal inspiratory and expiratory pressures and sniff nasal inspiratory pressure were improved in the experimental group when compared with the sham group (*p* < 0.05). However, there was no improvement in spirometric measurements when groups were compared (*p* > 0.05).

The prospective case-control study by Freitag et al. (2018) [45] investigated the effects of a sixteen-weeks respiratory muscle endurance training (RMET) on MG patients and compared the results with a control group. Eighteen patients with mild to moderate MG participated as the training group, and six patients served as controls. A modulation in the breathing pattern at rest with prolonged expiration was observed in the training group. In addition, the training group reported subjective improvements in MG symptoms, respiratory symptoms, and physical fitness. No significant changes were observed in the control group. 

#### 3.2.3. Balance Training

The prospective study by Wong et al. (2014) [46] aimed to determine if a 16 session workstation intervention consisting of balance strategy training (BST) could improve functional ability and balance in a group of seven individuals with MG. The quantitative myasthenia gravis score (QMG), timed up and go with cognitive task (TUG-cognitive), and foam with eyes closed (foam EC) achieved clinically significant improvements (>15%).

### 3.3. Summary of Clinical Findings

This review, including just two RCTs, one case-control study, and eight cohort studies in a total amount of 166 participants, reflects the paucity of research for rehabilitation of MG. Concerning clinical results, we found that physical training, both strengthening and resistive, enhanced functional ability and muscle force in MG patients, without causing adverse events. Equally good results were achieved with supervised home-based physical training. Respiratory muscle training, practiced using a threshold device or with normocapnic hyperpnea, was able to improve lung volumes and capacity in individuals with MG, and above all in those with moderate symptoms. In addition, we found that balance training enhanced vestibular function reducing the risk of falls. Finally, all the analyzed rehabilitative approaches contributed to reduce fatigue and to improving the quality of life of MG patients, especially when combined with psychological support. 

### 3.4. Clinical Heterogeneity

Clinical heterogeneity between selected studies was substantial. There was no mention of the diagnostic criteria used and of the MG classification (e.g., late onset, early onset, etc.). Furthermore, there was large variation in disease severity according to Osserman and Genkins, as well as in the participants’ mobility capabilities and respiratory muscle function. The applied experimental treatment protocols varied in terms of focus, intensity/dose, and duration. The outcome assessment was carried out with different and heterogeneous tools.

### 3.5. Methodological Quality

According to RoB 2, the RCTs by Fregonezi et al. and Aslan et al. received a high risk of bias judgment due to the lack of accurate information about the randomization process. The case-control study by Freitag et al. received a fair methodological quality score on the modified NOS because of the non-representativeness of cases, the inaccuracy concerning selection and definition of controls, and the incomplete data about exposure. The remaining cohort studies were all judged fair in agreement with the modified NOS; the non-representativeness of exposed individuals, the lack of comparability of cohorts, the incompleteness of follow-up, and the absence of accurate data about the outcome assessment were the most common problems.

### 3.6. Quality of Evidence

The level of evidence of the studies included in this review ranged from 2b to 4 on the OCEBM scale. Two studies were fair-quality RCTs with level of evidence 2b. One study was a fair-quality nonrandomized case-control trial with level of evidence 3b. The remaining studies were fair-quality cohort studies with level of evidence 4. In agreement with the OCEBM grades of recommendation, three of the studies were categorized as grade B and eight as grade C.

## 4. Discussion

MG is a chronic autoimmune disorder characterized by weakness of skeletal muscles. MG patients frequently present with multiple deficits (fatigue, diplopia, dysarthria, dyspnea, dysphagia, etc.) that require specific and coordinated multidisciplinary approaches. Rehabilitation alone or in combination with other forms of treatment can relieve or reduce symptoms for some people with MG. Nevertheless, it is well known that high intensity physical activity should be avoided in patients with MG because it increases muscular weakness. Finally, MG patients should find the optimal balance between physical activity and rest. This review systematically summarizes the best, up-to-date evidence from published studies for the effectiveness and safety of rehabilitation interventions for MG.

The main finding of this systematic review is that there is a critical lack of high-quality evidence for the effectiveness of various rehabilitation modalities for people with MG; although a spectrum of interventions is proposed, the evidence for many of these are limited due to a paucity of robust, methodologically strong studies.

The rehabilitative approaches most frequently evaluated in the selected studies were physical and respiratory training. Only one study analyzed balance training. Physical training, which includes aerobic, strength, and progressive resistance exercises, has proven to be an efficient strategy to improve functional outcomes (mobility, muscle strength, aerobic capacity), fatigue, physical performance, and quality of life in people with MG. The greatest benefits of physical training have been achieved in patients with a mild to moderate MG and practiced under a limited training intensity [36]. It has also been stated that general recommendations concerning physical exercise could be applied safely to patients with a well-regulated MG [38]. A study even showed that long-term physical activity could reduce the autoimmune response [38]. Furthermore, there was general agreement among selected studies that physical training is well tolerated by patients with MG and that the pathology does not deteriorate with physical activity [36,38,39].

Respiratory training has proven to be a very effective approach in the management of fatigable weakness and respiratory failure, both of which strongly limit the performance of daily activities in people with MG [13,41,42,43,47,48,49,50,51]. The benefits of respiratory training included not only a measurable improvement in respiratory muscle strength, in respiratory endurance, and in physical performance [41,42,43,44,45], but also a reduction in the incidence of several MG complications, like dyspnea [52]. Furthermore, sustained hyperpnea training was considered a more appropriate approach than respiratory strength training in people with MG, because it reduced significantly diaphragm and abdominal muscles fatigue [45]. In addition, respiratory training improved MG subjective symptoms, like fatigability. The effects of respiratory training can be explained by the fact that a long-term respiratory muscle endurance training reduces the patient’s respiratory rate. As a consequence, the work of breathing decreases the breathing reserve during physical activity and improves overall physical fitness. Respiratory training could be also practiced at home under the supervision of clinicians in order to enhance the conventional pharmaceutical therapy [45].

Balance training consists of exercises that target the sensorimotor system in order to improve function and reduce the risk of falls [46]. Additionally, balance training could also increase bone density, which can be reduced due to the sedentary lifestyle caused by muscle weakness and fatigue [53]. The positive effect of balance exercises on symptoms might be explained by an increase in the number of mitochondria within muscles, by the musculoskeletal mass building, and by the impulse to lactate degradation [54]. Consequently, this would lead to more efficient neuromuscular transmission, to the increase ability of muscles to cope with fatigue, and to improved strength and endurance [55]. Moreover, the increased visual-vestibular integration would lead to a better balance, since vestibular input has been suggested to play a key role in balance control [56,57]. 

Eventually, psychotherapy in combination with physical training (aerobic, stretching, and breathing strategies) could improve patient’s fatigue more than physical training alone [40]. Moreover, group therapy could modify a MG patient’s lifestyle since it helps to manage anxiety, depression, and social isolation [40].

The risk of bias in individual studies was very high due to the fair methodological quality of the selected studies. 

This review has several limitations: (1) the small number and the fair methodological quality of selected studies; (2) the heterogeneity of selected studies in terms of participants, interventions, and outcomes, resulting in the impossibility of carrying out a meta-analysis; and (3) the lack of publication bias assessment.

In conclusion, this review analyzed the evidence of published studies concerning the effectiveness and safety of rehabilitation in patients with MG. Despite the variety of rehabilitative approaches available, there is a lack of high-quality evidence for almost all strategies. Currently, no single reviewed approach can be recommended over another for the lack of cross-comparative studies, but there is some level of evidence for each individual intervention with regards to its specifically measured outcomes. According to the findings of this review, the best approach could be a multidisciplinary one that combines physical, respiratory, and balance training. If performed at moderate exercise intensity, such a multidisciplinary approach could improve symptoms, functional outcomes, and quality of life in MG patients. No side effects have been linked to physical training in individuals with MG. However, it is suggested to restrict it to patients with mild to moderate symptomatology (stage IIA and IIB of the disease). Moreover, MG patients’ awareness should always be promoted. In particular, patients should be advised to follow a home-based respiratory program, supervised by clinicians, which can help them to manage symptoms. Psychological support should be recommended, especially in-group sessions, in order to increase coping strategies.

More studies with robust methodology are still needed to justify the use of rehabilitation interventions in people with MG. Further research must also analyze type and intensity of rehab modalities, and cost-effectiveness of these interventions.

## Figures and Tables

**Table 1:** Search strategy.

Search	Query	Items Found
#1	Search (Myasthenia gravis)	18,642
#2	Search (Rehabilitation OR Rehab OR Rehabil [mesh])	641,763
#3	Search (Physical Therapy OR Physiotherapy OR Physical Therapy Modalities [mesh] OR Physical Ther Techniques [mesh])	351,211
#4	Search (#1 AND #2 AND #3)	75

**Table 2:** Summary of the articles included in the review.

Author and Year	Type of Study	Rehabilitation Approaches in the Experimental Group	Total Number of Participants	Main Results/Findings
Lohi et al., 1993 [36]	Cohort study	Strength resistive training program of 30 sessions over 10 weeks	11	A 23% increase in maximum voluntary muscle force in knee extension was observed on the trained side compared with the untrained one. Cohen’s d cannot be assessed due to the lack of means and standard deviations.
Weiner et al., 1998 [41]	Cohort study	Inspiratory and expiratory muscle training with a threshold muscle trainer, for 30′/day, 6 times a week, for 3 months	16	The maximal inspiratory muscle pressure, the forced vital capacity, the forced expiratory volume, and the dyspnea index score increased significantly in both groups (respectively *p* = 0.001, 0.005, <0.001, <0.001, <0.001, <0.001, <0.005, <0.001; Cohen’s d = 0.016, 0.02, 0.012, 0.02, 0.012, 0.016, 0.014, 0.02). The maximal expiratory muscle pressure increased significantly in group A (*p* < 0.05; Cohen’s d = 0.02) but remained unchanged in group B.
Fregonezi et al., 2005 [42]	RCT	Interval-based inspiratory muscle training combined with breathing retraining, three times a week for 8 weeks	27	The training group improved significantly compared to control group in maximal inspiratory pressure, maximal expiratory pressure, respiratory rate/tidal volume ratio, and upper chest wall expansion and reduction (respectively *p* = 0.001, 0.01, 0.05, 0.02, 0.04; Cohen’s d = 0.012, 0.02, 0.024, 0.014, 0.021).
Rassler et al., 2007 [43]	Cohort study	Respiratory muscle endurance training in form of normocapnic hyperpnea training at 50–60% of the maximal voluntary ventilation over 4–6 weeks	10	The training significantly increased respiratory endurance from 8.4 ± 0.9 min to 17.1 ± 1.3 min (*p* < 0.001; Cohen’s d = 0.014) and total ventilatory volume from 555 ± 87 L to 1081 ± 127 L (*p* = 0.004; Cohen’s d = 0.012).
Aslan et al., 2013 [44]	RCT	Respiratory muscle training performed by inspiratory and expiratory threshold loading methods	26	Maximal inspiratory and expiratory pressures, and sniff nasal inspiratory pressure were improved in the experimental group when compared with the sham group (respectively *p* = 0.002, 0.04, 0.04; Cohen’s d = 0.06, 0.01, 0.06).
Wong et al., 2014 [46]	Cohort study	16-session workstation intervention once or twice a week, consisting of balance strategy, strengthening, and endurance training exercises	7	Quantitative Myasthenia Gravis Score, Time Up and GO_cognitive_, and foam with eyes close achieved clinical significant improvement after intervention (>15%). Cohen’s d cannot be assessed due to the lack of means and standard deviations.
Rahbeck et al., 2016 [37]	Cohort study	20 training sessions during 8 weeks of either progressive resistance training or aerobic training	15	MG quality of life 15 and stair climb functional measure were improved in the PRT group when compared with the AT group (respectively *p* = 0.03 and 0.04; Cohen’s d = 0.04 and 0.06).
Westerberg et al., 2017 [38]	Cohort study	75-min session of supervised physiotherapy consisting of aerobic, muscle resistance, and balance training (two times weekly for 12 weeks)	10	CMAP amplitude biceps (mV), CMAP amplitude rectus femoris (mV), Six Minutes Walking Test (m), Biceps Curl (kg), Leg Extension (kg), and Muscle Range (%) achieved statistical significant improvement after intervention (respectively *p* = 0.002, 0.037, 0.002, 0.016, 0.0039, 0.019; Cohen’s d = 0.12, 0.02, 0.012, 0.6, 0.16, 0.05).
Freitag et al., 2018 [45]	Case-control	Long-term respiratory muscle endurance training based on normocapnic hyperpnea (4 weeks intensive training—5 × 30-min training sessions per week—followed by 12 months maintenance training—5 × 30-min training sessions over two weeks).	23	Respiratory endurance measured as time until exhaustion, the number of squats per minute, and the breathing patterns at rest with prolonged aspiration improved significantly after intervention (respectively +412%, +160%, +122%; Cohen’s d cannot be assessed due to the lack of means and standard deviations). The Myasthenia Gravis score improved from 0.67 ± 0.09 to 0.41 ± 0.1 (*p* = 0.004; Cohen’s d = 0.28). No significative changes were observed in the CG.
Westerberg et al., 2018 [39]	Cohort study	12 weeks supervised physical therapy regimen of aerobic and resistance strength training	11	After the training program, the rectus femoris muscle action potential, the rectus femoris muscle isometric force, and the rectus femoris muscle ultrasound thickness statistically improved (respectively, *p* = 0.016, 0.014, 0.098; Cohen’s d = 0.11, 0.29, 0.15). The 30-s chair stand test and the clinical Myasthenia Gravis composite score equally improved (respectively, *p* = 0.039, 0.043) but the Cohen’s d cannot be assessed due to the lack of means and standard deviations.
Farrugia et al., 2018 [40]	Cohort study	10-week program comprising physical and psychological interventions	10	There was a significant improvement in the Visual Analogue Fatigue Scale at the end of the program (*p* < 0.01 and Cohen’s d = 0.68). No clear improvement was noted in the other assessment tools.

CG: control group; RCT = randomized controlled trial; EG: experimental group; MG = myasthenia gravis; AT = aerobic training; PRT = progressive resistance training; CMAP: compound motor action potential.
